# A highly stretchable humidity sensor based on spandex covered yarns and nanostructured polyaniline

**DOI:** 10.1039/c7ra10474j

**Published:** 2018-01-03

**Authors:** Ya-Nan Guo, Zhi-Yuan Gao, Xiao-Xiong Wang, Li Sun, Xu Yan, Shi-Ying Yan, Yun-Ze Long, Wen-Peng Han

**Affiliations:** College of Physics, Qingdao University Qingdao 266071 China han_wenpeng@163.com; Industrial Research Institute of Nonwovens & Technical Textiles, Qingdao University Qingdao 266071 China

## Abstract

Stretchable sensors, as the important components of flexible electronic devices, have achieved progress in a variety of applications for monitoring physical or environmental conditions, such as sound, temperature, vibration, and pressure. However, it still remains a challenge to fabricate high performance stretchable humidity sensors. Herein, we present a novel stretchable humidity sensor, which was fabricated based on an ultrastretchable polyaniline composite fiber. Because of the composite fiber with a “twining spring” configuration (cotton fibers twining spirally around a polyurethane fiber) it maintains a stable electrical conductivity up to a strain of 200%. In addition, the conductivity of the composite fiber remains perfectly stable after 5000 cyclic stretching events of 200% strain. Incorporating the humidity sensitive properties of nanostructured polyaniline, the stretchable humidity sensor based on the composite fiber effectively maintains its humidity sensitivity at different elongations.

## Abstract 2

In this work, a highly stretchable humidity sensor based on spandex covered yarns and nanostructured polyaniline was fabricated, and maintains its humidity sensitivity well at different elongations.

## Graphical abstract

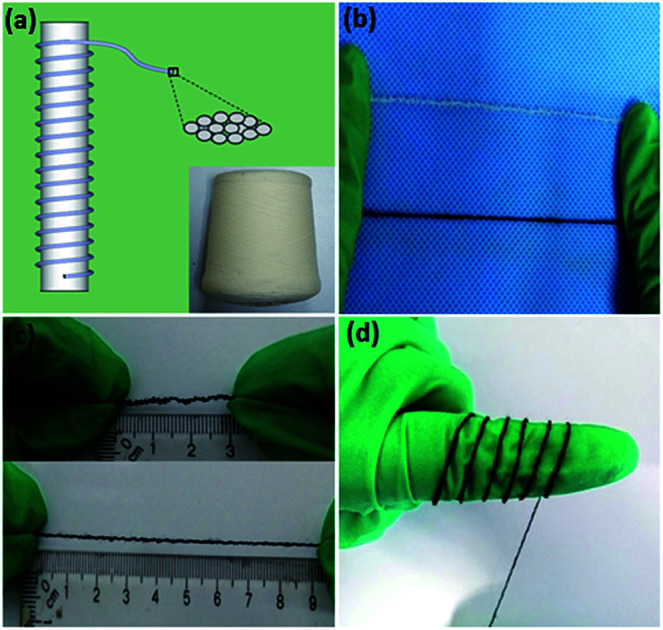

## 1 Introduction

In recent years, stretchable electronics as an emerging and interesting research scope has motivated intensive efforts from materials scientists and engineers. This new class of electronics has opened up a wide variety of extraordinary application fields, including stretchable displays,^1,2^ wearable devices,^3^ electronic eye cameras,^4^ stretchable energy-storage devices,^5,6^ and implantable devices for human health monitoring.^7^ As an important component of integrated electrical devices, sensors perform the role of signal-input for responding various external stimulations. With the rapid development of stretchable electronics, it has become increasingly urgent to develop a new generation of stretchable sensors.

Polyaniline (PANI), as an environmentally stable conducting polymer, has been widely studied due to its unique properties, such as simple preparation and doping procedure, relatively high conductivity and low cost. To date, many sensors based on PANI material have been fabricated successfully for application in different fields, such as chemical sensor,^8,9^ gas sensor,^10,11^ bio-sensor,^12,13^ and strain/pressure sensor,^14^ Recently the importance of humidity sensing has been well understood and a great deal of research has focused on the development of humidity sensitive materials because humidity sensors have gained increasing applications in industrial processing and environmental control. It is verified that PANI is sensitive to humidity because its conductivity changes in the presence of water vapor due to the “proton effect”.^15^ Numerous studies also have reported^16–21^ humidity sensors, constructed based on PANI or its derivative materials. In general, chemically prepared PANI materials are usually powdery and have poor mechanical properties. To fabricate stretchable and flexible devices based on PANI material, the common approach is to prepare functional composites by incorporation of PANI into a rubbery polymer matrix through blending.^22,23^ However, simultaneously increasing stretchability and electronic conductivity is challenging because these properties are competitive, and the conductivity of those composites decreased significantly under strain tension. An alternative strategy is to use the stretchable materials as a fiber-core or substrate and cover them with PANI.^14,24^ In order to increase stretchability, a patterned poly(vinylidene fluoride) (PVDF) nanofibrous membrane was prepared *via* electrospinning and coated with PANI by *in situ* polymerization.^14^ Although the patterned nanofibrous membrane could be stretched up to 110%, the conductivity decreased significantly with the increase of deformation. As mentioned above, these stretchable materials based on PANI are more suitable to be considered for assembling strain/pressure sensors than humidity sensors because the conductivity of the material to fabricate a stretchable humidity sensor must remain stable when tensile deformation occurs. Recently, Lim *et al.* prepared different types of stretchable composite materials and discussed the resistance change depending on applied strain.^24–26^ They reported a stretchable humidity sensor based on a wrinkled polyaniline nanostructure. However, the performance of the sensor was not stable, particularly at low level humidity and a high elongation range above 30%.

Until now, it still remains a challenging task to develop a facile, cost-effective, and scalable way to prepare highly stretchable conductive materials, which are suitable for fabricating stretchable humidity sensors. In this study, we report the facile fabrication of highly stretchable composite conductive fibers based on spandex covered yarns (SCYs) and nanostructured PANI for the first time. The composite fiber comprises a selected SCY, serving as an elastic scaffold and nanostructured PANI, formed by *in situ* polymerization, serving as the conducting component. Since the unique spiral configuration of the SCY preserves extremely large prestrain, no actual elongation of the conducting component takes place when the composite fiber is stretched. It can be stretched up to 200% and maintains a stable conductivity at different elongation. The composite fiber also possesses superb cyclic performance without making any compromises on overall performance after 5000 stretching events of 200% strain and the conductivity changes only slightly due to the variation of ambient temperature from 20 to 40 °C. The stretchable humidity sensor based on the composite fiber was fabricated, featuring with good humidity-sensitivity, fast response, and good repeatability. In addition, the characteristics of the textiles allow them to be employed in the health, leisure and sports industries.

## 2 Experiments

### 2.1 Fabrication of SCY–PANI fibers

**Fig. 1:**
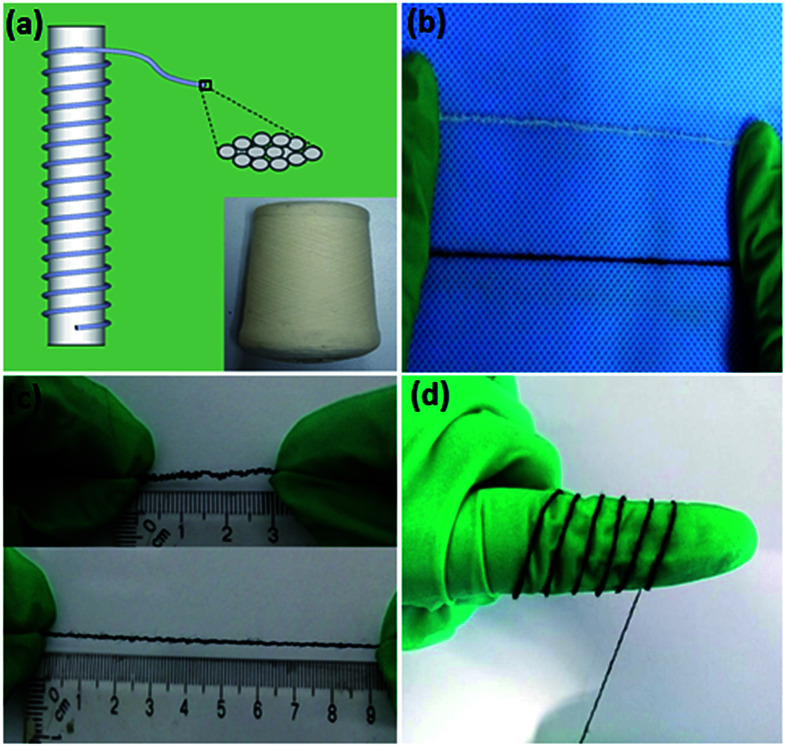
(a) Structural representation of the SCY. The inset is the image of the SCY spindle. (b) Comparison of the SCY (white) and the SCY–PANI fiber (dark green). (c) A SCY–PANI fiber of 3 cm was stretched up to 9 cm by hand. (d) The flexible SCY–PANI fiber conformed well to a human finger, demonstrating its possible utilization in wearable electronics.

Some SCYs were taken directly from the SCY spindle (the inset in Fig. 1a), which is widely and easily accessible in the textiles market. Then, the SCYs were prepared to be the framework of SCY–PANI fibers after being cleaned in ethanol with ultrasonic treatment for about ten minutes and dried in air. PANI was prepared by the solution polymerization of aniline with ammonium persulphate (APS) as an oxidant and 5-sulfosalicylic acid (SSA) as a dopant.^14,27^ First, an aqueous solution of APS (used as an oxidant) was prepared by dissolving 9.1 g of APS in 100 mL of deionized water. Further, 3.72 g of aniline was dissolved in 100 mL of aqueous solution, which contains 5.08 g of SSA. Then, the APS solution was mixed dropwise to start the oxidation. In order to obtain the SCY–PANI fibers, the as-prepared SCYs were immersed in the blended solution, and the obtained mixture was kept steady for 12 h with the reaction temperature at about 4 °C. In the end, the SCYs turned from the original milky white colour to dark green, along with some dark green precipitate formed in the reaction vessel due to small amount of PANI prepared in the polymerization process. Then, the SCYs were taken out and washed with distilled water several times to remove any of the oxidant present till the filtered water became colourless. Finally, the prepared fibers were dried in a vacuum oven at 45 °C for 48 h.

### 2.2 Preparation of humidity sensors based on the SCY–PANI fibers

**Fig. 2:**
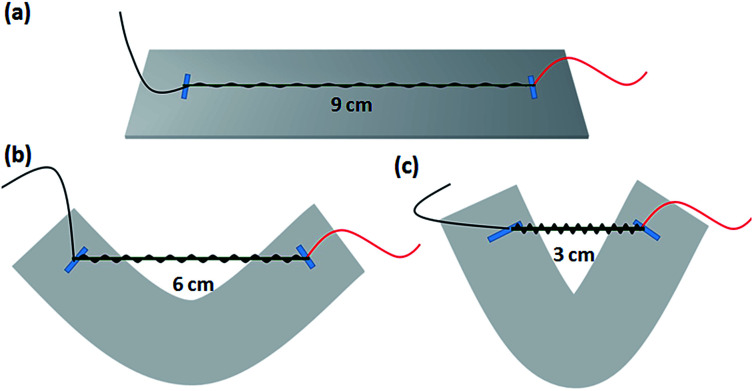
Schematic diagrams of the SCY–PANI fiber sensor, which under the different strains (a) 200%, (b) 100%, and (c) 0%.

For fabricating the SCY–PANI fiber humidity sensor, the prepared SCY–PANI fiber was cut into small segments of 3 cm. An insulating and soft flake of 2 × 10 cm^2^ in size was chosen as the substrate, and the two ends of the fiber segment which in completely stretched state were fixed on the substrate with doublesided tape. Therefore, the length of the fiber segment can be changed from the completely stretched state to original length by pressing the ends of the substrate as shown in Fig. 2. Finally, two electrodes were prepared on both ends of the SCY–PANI fiber using quick drying and highly conducting silver paste to enhance the contact between the SCY–PANI fiber and copper wires.

### 2.3 Measurements

**Fig. 3:**
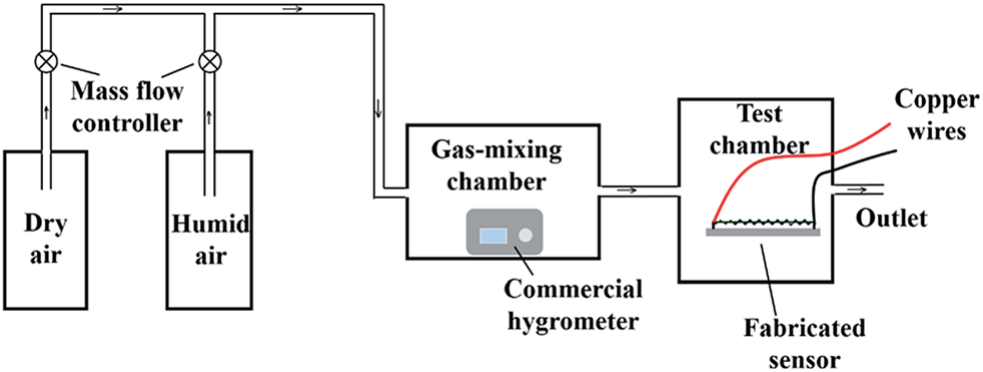
Schematic diagram of the system to measure the humidity sensitive properties of the SCY–PANI fiber sensor. The humidity of the air is firstly measured by using a commercial hygrometer.

Morphologies of the SCY–PANI fibers were observed using a Phenom ProX scanning electron microscope (SEM) with an accelerating voltage of 15 kV. Humidity sensitive properties of the sensors were investigated by recording their electrical response to relative humidity. A schematic diagram of the humidity-sensing measurement system in our laboratory is displayed in Fig. 3. We use humid air as a target gas and air flow is controlled by a mass flow controller. Humid air flows into a gas-mixing chamber, where the humid air is mixed with the dry air to control the relative humidity. The relative humidity of the air obtained by the mixing is first measured by a commercial hygrometer. Then, the air is injected to the test chamber. Because the four-point method is inconvenient for the real time electrical response testing of the SCY–PANI fibers under mechanical deformation, the electrical response of the sensors was measured by a two-probe method with a Keithley 6487 high resistance meter system, which connects to the sensor using the copper wires (Fig. 3).

## 3 Results and discussion

### 3.1 Basic properties of the sensors

**Fig. 4:**
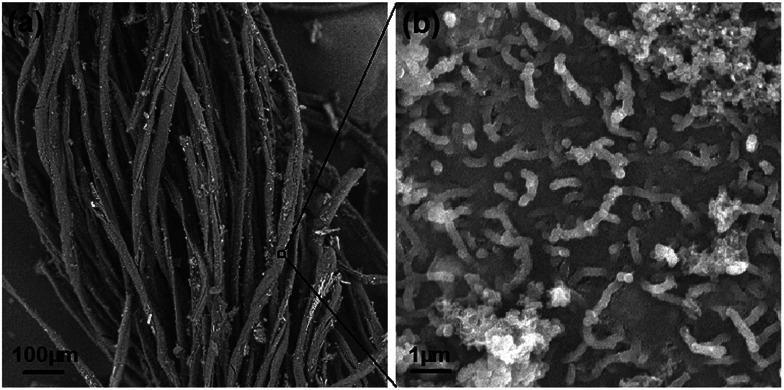
(a) SEM image of the SCY–PANI fiber. (b) Enlarged SEM image for the surface of the SCY–PANI fiber, PANI nanostructures can be seen clearly.

As a type of cotton/spandex yarn, the SCY is inexpensive and widely used in the textile industry. Typically, the high-elastic polyurethane monofilament served as the core fiber and was tightly wrapped by a cotton yarn, which consists of a large amount of cotton fibers, such as a compressed spring (Fig. 1a). This type of SCY is carefully selected for the reason that it combines perfectly the superb elasticity provided by the core fiber (polyurethane monofilament) and increased specific surface area by the cotton yarn, which is key to the efficient polymerization of PANI. Moreover, because the unique spiral configuration of the cotton yarn preserves extremely large prestrain, no actual elongation of the cotton yarn takes place when the SCY is stretched. As shown in Fig. 1b, the as-prepared SCY–PANI fiber turned to dark blue from the milk white colour of SCY due to the PANIs produced after the polymerization process. The PANIs are distributed evenly on the surface of the cotton fibers, which is confirmed by the SEM image in Fig. 4b. Because of the special spiral structure (Fig. 4a), the SCY–PANI fiber maintained high elasticity that would not fracture at a strain up to 200% (Fig. 1c) and can conform well to arbitrary curvilinear surfaces, such as a finger as shown in Fig. 1d. This demonstrates that the SCY–PANI fibers have a potential application in wearable devices.

**Fig. 5:**
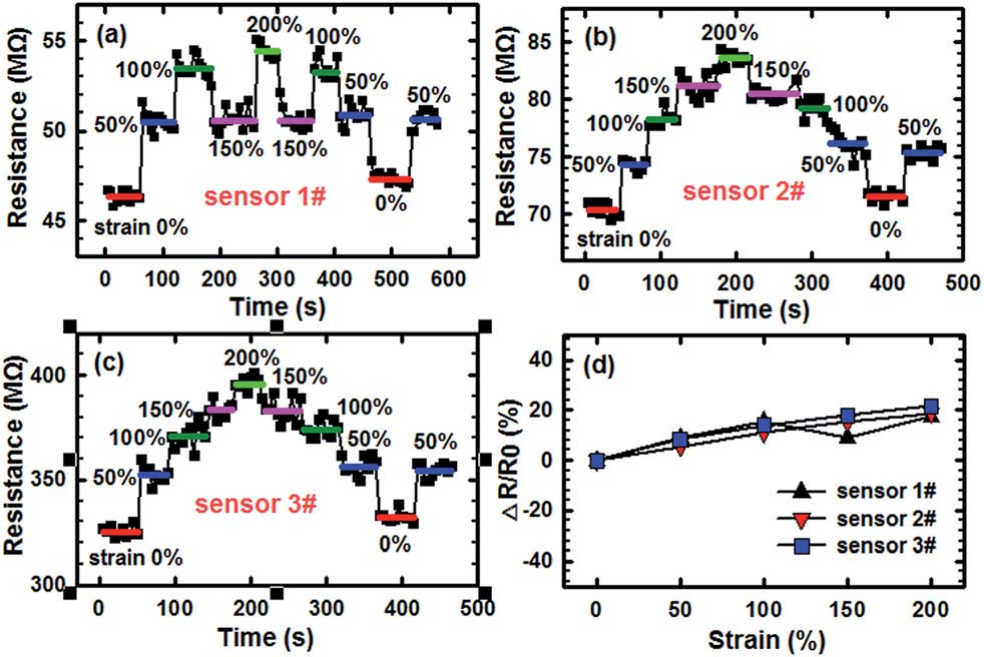
The dependences of response on the tensile strain for the different sensors (a) sensor 1#, (b) sensor 2#, and (c) sensor 3# at ambient conditions (20 °C, 27% RH). (d) Dependences of the resistances of different sensors on strain. *R*_0_ is the initial resistance at original length and Δ*R* is the variation of resistance after being stretched.

The key to realizing stretchable electronics is the simultaneous incorporation of excellent mechanical robustness and relatively stable electronic performance. In particular, for fabricating the stretchable humidity sensors, the material must maintain a stable electrical conductivity at different elongations. The effects of imposed severe deformation, such as stretching and bending, on the electrical conductivity of the sensors were investigated herein. In this investigation, three sensors were fabricated based on the SCY–PANI fiber segments, which were selected at random and marked as 1#, 2#, and 3#. Fig. 5(a–c) display the resistances of the different sensors recorded during uniaxial stretching up to a strain of 200%. The profile of resistance change is different for each sensor because they are not made by a precise fabrication operation. However, the results demonstrate that the maximum variation of resistance caused by deformation of the three fibers is only 17.39%, 18.80%, and 21.68% of the initial resistance as shown in Fig. 5d. To further illustrate this point about the capability of the SCY–PANI fiber to maintain the conductivity after being stretched, the figure of merit (−Δ*R*/*R*_0_)/*S*, where *R*_0_ is the initial resistance and Δ*R* is the variation of resistance after being stretched to a strain of *S*, was calculated for different strains. Clearly, the smaller the figure of merit, the higher the capability for the stretchable fiber to preserve its conductivity. Recently, another study reported an ultrastretchable composite fiber, which also used the SCY as the fiber core and covered with silver nanowires as the conducting component.^28^ The figures of merit for this case are 0.251 and 0.199 at strains of 100% and 200%, respectively. In our study, the figures of merit for the sensor 1# are 0.173, 0.152, 0.058, and 0.087 at strains of 50%, 100%, 150%, and 200%, respectively, indicating the superior stability of conductivity during the deformation.

**Fig. 6:**
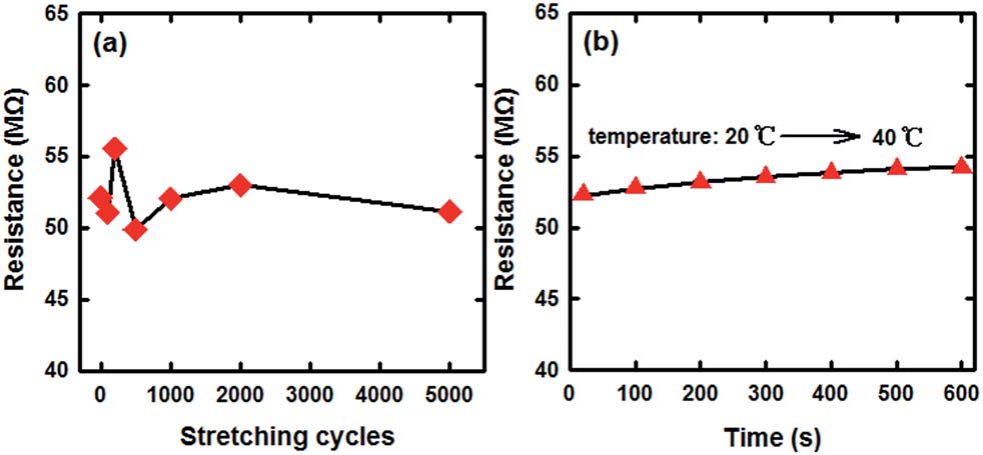
(a) The resistances of the sensor 1# after increasing stretching cycles (one cycle: from strain of 0–200% and back to 0%) which were measured at strain of 100%, 20 °C and 27% RH. (b) The dependence of response on the temperature for the sensor 1# at strain of 100% and 27% RH.

A practical stretchable humidity sensor must possess stable performance. Because there was an acidic environment, the mechanical properties became poor during the process of producing SCY–PANI fibers. By adjusting the weights of SSA in the reaction process we can obtain SCY–PANI fibers that have relatively good mechanical properties. In our study, no significant changes in appearance and mechanical properties of the SCY–PANI fibers were observed after 5000 cyclic stretching events of 200% strain. We further undertook a cyclic stretching test of the sensor so as to evaluate the stability of the electrical property, which was a crucial factor from the viewpoint of practical applications. Fig. 6a illustrates the resistance of the sensor 1# after cyclic stretching at a strain of 100%. From the results, the resistance of the sensor is stabilized in the vicinity of 52 ± 3 MΩ. As an added bonus, the electrical conductivity of the sensor has superior temperature stability. As shown in Fig. 6b, the resistance of the sensor at a strain of 100% slightly changed in the process of temperature rise from ambient temperature (20 °C) to 40 °C. All these qualities listed above undoubtedly make our SCY–PANI fiber a superior candidate material for fabricating stretchable sensors.

### 3.2 Humidity sensitive properties

**Fig. 7:**
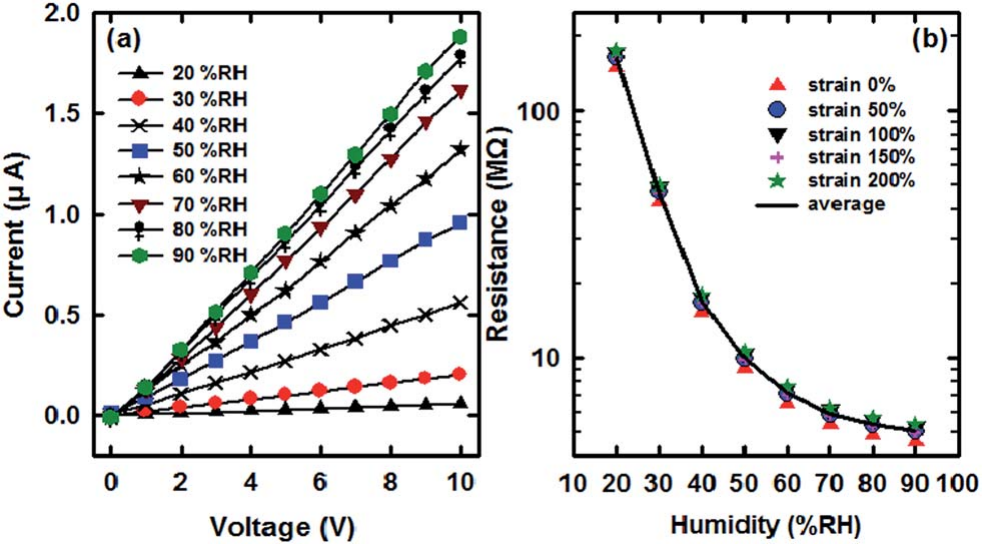
Humidity sensitive properties of the stretchable sensor 1# based on the SCY–PANI fiber. (a) The voltammetry curves of the stretchable sensor 1# under different % RHs which were measured at strain of 100% and 20 °C. (b) Humidity responses of the stretchable sensor 1# at strains of 0%, 50%, 100%, 150%, and 200%, respectively. The solid line is the average of the different resistances caused by the various elongations.

It is reported that water increases the electrical conductivity of PANI through an increase in the interchain electron transfer and/or by increasing the mobility of dopant ions.^29^ Due to polymerization by strong oxidant (APS) and the un-bonded electron pair on the nitrogen atom, PANI has a chain with the protonated reduced (NH^2+^) and oxidized form (NH^+^). As evidenced by the NMR experiment, the proton transfer from or to the polymer takes place in the presence of water molecules.^30^ It is because of the existence of these protons, the electron transfer from NH^2+^ to the NH^+^ becomes easier. Thus, absorbed water plays an important role in the conductivity.^31^ Variation of voltammetry curves of as-prepared humidity sensor at the different relative humidity (% RH) are presented in Fig. 7a. Furthermore, the corresponding resistances of each % RH are shown in Fig. 7b, which shows that the observed resistance decreases with % RH. In addition, under the different fixed % RHs, the humidity sensor maintained a stable sensitivity at the different elongations, particularly when the % RH is less than 60. When the average of resistances at different strains was used to calibrate the environment humidity as a standard value, the % RH errors are ±1, ±1, ±2, ±3 and ±4 under the various fixed % RHs (20, 30, 40, 50 and 60% RH), respectively. However, once the % RH goes beyond 70, the % RH error exceeds 10 as shown in Fig. 7b. The reason is because the cotton fiber has good hygroscopicity, sufficient amount of water molecules covered the PANI surface once the % RH goes beyond 70.

**Fig. 8:**
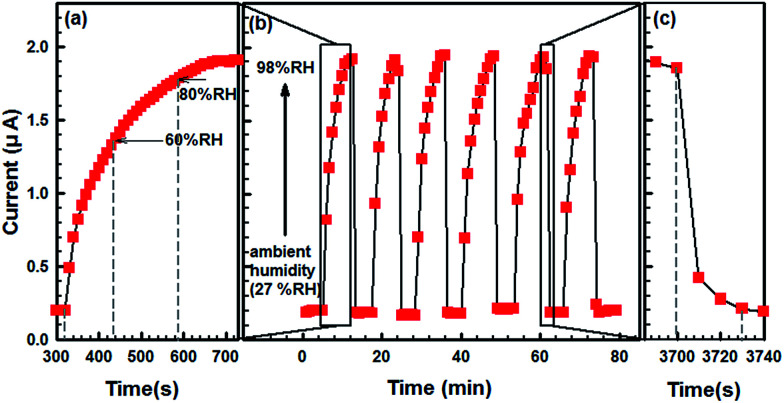
Response transients of the stretchable sensor 1#, (a) and (c) are the process details of humidification and desiccation which were shown in (b).

Typical response transients of as-prepared humidity sensor obtained from SCY–PANI fiber during cycle tests are shown in Fig. 8b. It is clearly observed that the sensor revealed good repeatability. Moreover, the response time of the SCY–PANI fiber between 27% RH (ambient humidity) and 98% RH was found to be 400 s and 30 s for humidification and desiccation processes, respectively, as estimated from the response transients shown in Fig. 8a and c. The response time is a crucial parameter for evaluating the performance of a humidity sensor. In our study, the sensor does not exhibit a rapid response time, particularly during humidification. However, this does not mean that the SCY–PANI fiber is not a promising material for the humidity sensor. As shown in Fig. 8a, the response time for the % RH to reach 60 was estimated at 116 s, which accounts for only over a quarter of the entire humidification process. Thus, all the above results suggest that the SCY–PANI fiber is a promising material for the stretchable humidity sensor, particularly when the % RH is not higher than 60.

## 4 Conclusions

In summary, we demonstrated the highly stretchable humidity sensor, which was fabricated based on a PANI composite fiber. The fabricated sensors stably maintained their good performance up to a strain of 200%, which benefited from the unique “twining spring” configuration of the composite fibers and the humidity sensitive properties of the PANI. In addition, the PANI composite fiber possesses a superb cyclic property and has the advantages of low cost, simple preparation, and scale production. We believe that the advantages of our fabrication strategy can be extended to environment and health monitors, sensory skins for robotics and many other applications of stretchable electronics.

## Conflicts of interest

There are no conflicts to declare.

## Supplementary Material
